# Effect of Alkaloid Extract from African Jointfir (*Gnetum africanum*) Leaves on Manganese-Induced Toxicity in *Drosophila melanogaster*

**DOI:** 10.1155/2018/8952646

**Published:** 2018-12-30

**Authors:** Ganiyu Oboh, Opeyemi Babatunde Ogunsuyi, Olatunde Isaac Awonyemi, Victor Ayomide Atoki

**Affiliations:** ^1^Department of Biochemistry, Federal University of Technology, P.M.B. 704 Akure, Nigeria; ^2^Department of Biomedical Technology, Federal University of Technology, P.M.B. 704 Akure, Nigeria; ^3^Central Research Laboratory, Federal University of Technology, P.M.B. 704 Akure, Nigeria

## Abstract

Metal-induced toxicity in fruit fly (*Drosophila melanogaster)* is one of the established models for studying neurotoxicity and neurodegenerative diseases. Phytochemicals, especially alkaloids, have been reported to exhibit neuroprotection. Here, we assessed the protective effect of alkaloid extract from African Jointfir (*Gnetum africanum)* leaf on manganese- (Mn-) induced toxicity in wild type fruit fly. Flies were exposed to 10 mM Mn, the alkaloid extract and cotreatment of Mn plus extract, respectively. The survival rate and locomotor performance of the flies were assessed 5 days posttreatment, at which point the flies were homogenized and assayed for acetylcholinesterase (AChE) activity, nitric oxide (NO), and reactive oxygen species (ROS) levels. Results showed that the extract significantly reverted Mn-induced reduction in the survival rate and locomotor performance of the flies. Furthermore, the extract counteracted the Mn-induced elevation in AChE activity, NO, and ROS levels. The alkaloid extract of the African Jointfir leaf may hence be a source of useful phytochemicals for the development of novel therapies for the management of neurodegeneration.

## 1. Introduction

Manganese (Mn) is essential for a wide array of biochemical processes in the body [1]. However, exposure to excessive level of Mn through, for example, occupational means can induce neurodegenerative diseases with pathophysiological features similar to Parkinson's disease [2]. Neurodegenerative diseases are pathologies of multiple causative factors; examples include Alzheimer's disease (AD) and Parkinson's disease (PD). Studies reveal that these diseases are characterized by decline in neurotransmitters associated with the brain, such as acetylcholine and neuroactive amines, as well as oxidative stress caused by excessive levels of metal ions in the brain [3]. It has therefore become essential to develop holistic therapeutic approach for preventing and managing these diseases by focusing attention on the different risk factors. Cholinergic neurons make use of the neurotransmitter acetylcholine which is metabolized by cholinesterase enzymes [4–6]. Therefore, cholinesterase inhibitors such as galantamine and donepezil have been used as therapeutic intervention for AD [6, 7]. Over the years, cholinesterase and monoamine oxidase inhibitors have been used as therapeutic approach for managing these diseases. These interventions have, however, been shown to pose serious adverse effects. Therefore, the importance of complementary/alternative dietary/medicinal interventions as a managerial and preventive approach cannot be overemphasized.

The fruit fly (*Drosophila melanogaster)* has gained a lot of use as an alternative animal model for biomedical research, especially for unraveling the molecular mechanisms behind several human diseases. Furthermore, studies have shown that up to 75% of the human disease-causing genes are conserved in *Drosophila* [8]. The similarity between human and Drosophila genomes is not only limited to genetic elements but also includes the relationship between them, with numerous examples of conserved biological mechanisms [9]. Metal-induced toxicity in *D. melanogaster* is an established model for studying neurotoxicity and neurodegenerative diseases. Previous studies have used metals such as Fe, Al, Cu, and Mn to induce neurotoxicity as models of neurodegeneration in *D. melanogaster*; Fe-, Cu-, and Mn-induced toxicity have been linked with PD and Parkinsonism [10], while Al is used to model Alzheimer-like pathology in *D. melanogaster* [11]. Specifically, the hallmark of Mn-induced neurotoxicity in *D. melanogaster* includes reduction in life span and locomotor performance, increased ROS generation, and impaired cholinergic and doperminergic systems [12–14].

Plant-based bioactive phytochemicals such as polyphenols and alkaloids have shown promising neuroprotective potentials in both *in vitro* [15] and *in vivo* rat [16, 17] and *drosophila* models [18, 19]. Green leafy vegetables form a major constituent of local diets and are the major sources of plant bioactive phytochemicals. They are desired not only for their nutritional benefits but also for their medicinal properties as reported in folklore. Notable among them is African Jointfir (*Gnetum africanum)*. African Jointfir (AFJ) is a leafy vegetable desired in different African countries and especially south-eastern Nigeria (where it is commonly referred to as “Okazi”) for its nourishment and medicinal properties [14, 20, 21]. The leaves are reported to be abundant in alkaloid phytochemicals [22] and has been used traditionally for treatment of several diseases such as fever, ulcer, and diabetes [23]. The leaves are consumed as spice and in preparation of soups and stews [24]. Furthermore, the hypolipidermic, hyperglycemic, anti-inflammatory, and antioxidant properties of this vegetable has been previously reported [25, 26]. This study, therefore, evaluated some of the biochemical mechanisms behind the protective properties of alkaloid extract of AFJ against manganese-induced neurotoxicity model in *D. melanogaster.*

## 2. Materials and Method

### 2.1. Sample Collection and Extraction of Crude Alkaloids Compounds

Fresh sample of African Jointfir (*Gnetum africanum)* leaves was sourced from local market in Akure, (South West) Nigeria, during the raining season (May) of 2017. The sample was identified and authenticated at the Department of Biology, Federal University of Technology, Akure, Nigeria (identification number: FUTA/BIO/404). The leaves were carefully separated, rinsed under running tap water, and dried for twenty days at room temperature (under shade) to constant weight. The pulverized sample was kept in an air-tight container prior the extraction of the alkaloids.

### 2.2. Reagents

Chemical reagents such as acetylthiocholine iodide, thiobarbituric acid, sulphanilamide, reduced glutathione, DEPPD, DMSO, DPPH, trichloroacetic acid, and sodium acetate were sourced from Sigma-Aldrich (now Merck KGaA, Darmstadt, Germany). Hydrogen peroxide, methanol, acetic acid, hydrochloric acid, aluminium chloride, potassium acetate, sodium dodecyl sulphate, iron (II) sulphate, manganese chloride, potassium ferrycyanide, and ferric chloride were sourced from BDH Chemicals Ltd., (Poole, England). Except stated otherwise, all other chemicals and reagents were of analytical grades and the water was glass distilled.

### 2.3. Fruit Fly *(Drosophila melanogaster)* Culturing

Wild type fruit fly (Harwich strain) stock culture (originally from the National Species Stock Centre (Bowling Green, OH, USA)) was obtained from the Drosophila Laboratory, Department of Biochemistry, University of Ibadan, Oyo State. The flies were maintained and reared on a normal diet made up of corn meal medium containing 1% *w/v* brewer's yeast and 0.08% *v/w* nipagin at room temperature under 12 h dark/light cycle conditions in the Drosophila Research Laboratory, Functional Foods and Nutraceutical Unit, Federal University of Technology, Akure, Nigeria. All the experiments were carried out with the same *D. melanogaster* strain.

### 2.4. Alkaloid Extract Preparation

Alkaloid extract of the sample was prepared according to the method of Harborne [27], with slight modifications [28]. This involved extracting 10 g of the samples in 100 mL of 10% alcoholic-acetic acid for 24 hours. The mixture was subsequently filtered to obtain clear filtrate. The filtrate was concentrated under a vacuum at 45°C in a rotary evaporator (Laborota 4000 Efficient, Heidolph, Germany), which was followed by NH_3_OH precipitation. The precipitate was collected as the crude alkaloid extract, dried thoroughly at 45°C and stored in the refrigerator at 4°C for all subsequent analysis. The yield of the extract obtained was 95 mg/g of dried leaf sample of African Jointfir. The extracts were dissolved in 1% DMSO for all subsequent analysis.

### 2.5. In Vitro Analysis

#### 2.5.1. Cholinesterase Activity Assay

The effect of the alkaloid extract on AChE activity was assessed by the modified Ellman colorimetric method [29]. The reaction consisted of 135 *μ*L of distilled water, 20 *μ*L of 100 mM sodium phosphate buffer (pH 8.0), 20 *μ*L of 10 mM DTNB, fly homogenate in 0.1 M phosphate buffer (pH 8.0) (see below for details of tissue homogenate preparation), appropriate dilutions of extract, and 20 *μ*L of 8 mM acetylthiocholine iodide as initiator. The reaction was monitored for 5 min (15 s intervals) at 412 nm using a spectrophotometer. A negative control assay was conducted to include the extract and homogenate, without the substrate. The AChE activity was thereafter expressed as percentage inhibition of the reference (homogenate AChE activity in the absence of the extract).

#### 2.5.2. Free Radical Scavenging Ability

The ability of the extract to scavenge free radicals using the DPPH model was assessed by the method of Gyamfi et al. [30] as previously reported [31] in a reaction mixture consisting of the extract (0-1 mL) and 1 mL of 0.4 mM methanolic-DPPH solution. This was followed by incubation in the dark for 30 min, and the absorbance was measured at 516 nm. The radical scavenging ability was expressed as percentage of the reference (reaction mixture excluding the extract).

#### 2.5.3. Iron Chelation Assay

The chelating ability of the extract against iron was determined using the method of Puntel et al. [32]. An aliquot of 150 *μ*L of 500 *μ*M FeSO_4_ which serve as the iron source was reacted with 168 *μ*L 0.1 M Tris–HCl (pH 7.4), 218 *μ*L saline, and the extract (0–25 *μ*L). The reaction mixture was incubated for 5 min, before addition of 13 *μ*L 0.25% 1, 10-phenanthroline (*w/v*). The absorbance was subsequently measured at 510 nm in a spectrophotometer. The Fe^2+^ chelating ability was expressed as percentage of the reference (reaction mixture excluding the extract).

### 2.6. In Vivo Analysis

#### 2.6.1. Experimental Design

Harwich strain of *D. melanogaster* (both gender, 3–5 days old) was divided into 5 groups containing 50 flies each (*n* = 3). Group 1 was placed on a normal diet (without alkaloid), while groups 2–4 were placed on a diet containing 10 mM Mn (sourced as MnCl_2_), Mn + AFJ alkaloid extract (final concentration of 2.5 mg/g of diet), and AFJ alkaloid extract alone, respectively. The flies were exposed to these treatments for 20 days and maintained at ambient temperature before being used for different assays. The choice of dose for Mn was based on preliminary survival study (data not shown) in which flies were exposed to varying concentrations of Mn (2–10 mM) supported by previously published data [14]. A preliminary study was conducted to ascertain that the dose of AFJ extract chosen and the vehicle (1% DMSO) showed no significant mortality to the flies (data not shown).

#### 2.6.2. Lethality Response

The flies were observed daily for the incidence of mortality, and the survival rate was determined by counting the number of dead flies, while the survivors were transferred to a freshly prepared diet weekly. The data were subsequently analysed and plotted as cumulative mortality and percentage of live flies after the treatment period [13].

#### 2.6.3. Measurement of Locomotor Performance by Negative Geotaxis Assay

The negative geotaxis assay was used to evaluate the locomotor performance of flies [33]. In brief, after the treatment period of five days, the flies from each group were briefly immobilized in ice and transferred into a clean tube (11 cm in length and 3.5 cm in diameter) labelled accordingly. The flies were initially allowed to recover from immobilization for 10 min and thereafter were tapped at the bottom of the tubes. Observations that were made for the total number of flies that crossed the 6 cm line within a period of 6 s were recorded. The results are expressed as percentage of flies that escaped beyond a minimum distance of 6 cm in 6 s during three independent experiments.

#### 2.6.4. Preparation of Tissue Homogenate

The flies were immobilized in ice and homogenized in 0.1 M phosphate buffer, pH 7.4. The resulting homogenates were centrifuged at 10,000 × g at 4°C for 10 minutes in a Kenxin refrigerated centrifuge Model KX3400C (KENXIN Intl. Co., Hong Kong). Subsequently, the supernatant was separated from the pellet into labelled Eppendorf microtubes and used for various biochemical assays.

### 2.7. Bioassays

#### 2.7.1. Acetylcholinesterase (AChE) Activity Assay

AChE activity was assayed according to the method of Ellman [34], with slight modifications. The reaction mixture was made up of 195 *μ*L of distilled water, 20 *μ*L of 100 mM sodium phosphate buffer (pH 8.0), 20 *μ*L of 10 mM DTNB, 5 *μ*L of homogenate, and 20 *μ*L of 8 mM acetylthiocholine (as initiator). Thereafter, reaction was monitored for 5 minutes (15-second intervals) at 412 nm. The AChE activity was thereafter calculated and expressed as mmolAcSch/h/mg protein.

#### 2.7.2. Total Reactive Oxygen Species (ROS) Level

Total ROS level in the whole fly tissue homogenates was estimated as H_2_O_2_ equivalent according to a previously reported method [35], with slight modifications. The reaction mixture consist of 50 *μ*L of tissue homogenate, 1400 *μ*L of 0.1 M sodium acetate buffer (pH 4.8), and 1000 *μ*L of reagent mixture of 6 mg/mL DEPPD and 4.37 *μ*M of FeSO_4_ dissolve in the sodium acetate buffer (1 : 25). The reaction was incubated at 37°C for 5 min, followed by absorbance measurement at 505 nm in a spectrophotometer. ROS levels were estimated from an H_2_O_2_ standard calibration curve and expressed as unit/mg protein, where 1 unit = 1 mg H_2_O_2_/L.

#### 2.7.3. Measurement of Nitric Oxide (NO)

NO content in the whole fly tissue homogenate was estimated using the Greiss reagent spectrophotometric based method [36] with slight modifications. Briefly, the reaction mixture consist of 150 *μ*L of tissue homogenate, 50 *μ*L of distilled water, and 600 *μ*L of Greiss reagent (0.1% *N*-(1-naphthyl)-ethylenediamine dihydrochloride, 1% sulfanilamide, and 2.5% phosphoric acid). This was followed by 10 min incubation at room temperature in the dark and absorbance measurement at 540 nm. The concentration of nitrite and nitrate as a measure of NO level was determined from the sodium nitrate standard curve and expressed as *μ*mol of NO/mg protein.

#### 2.7.4. Determination of Total Protein

The Bradford method [37], with bovine serum albumin (BSA) as standard, was used to quantify the total protein content of fly homogenates.

### 2.8. GC-MS Characterization

A qualitative characterization analysis of possible compounds present in AFJ was carried out using GC-MS (using scan mode) as previously reported [28] with slight modifications. Briefly, an aliquot of samples (500 mg) was dissolved in 10 mL of methanol. Thereafter, the analysis was performed using 7890A gas chromatograph coupled to 5975C inert mass spectrometer with electron-impact source (Agilent Technologies). The stationary phase of separation of the compounds was HP-5MS capillary column coated with 5% phenyl methyl siloxane (30 m length × 0.32 mm diameter × 0.25 *μ*m film thickness) (Agilent Technologies). The carrier gas was helium used at a constant flow of 1.0 mL/min at an initial nominal pressure of 19.6 MPa and average velocity of 33.425 cm/sec. An aliquot of the samples (1 *μ*L) was injected in splitless mode at an injection temperature of 110°C. Purge flow was 3 mL/min with a total flow of 11.762 mL/min; gas saver mode was switched on. Oven was initially programmed at 110°C (2 min) then ramped at 10°C/min to 200°C (2 min) then 5°C/min to 280°C (9 min). Run time was 38 min with a 3 min solvent delay. The mass spectrometer was operated in electron ionization mode with ionization energy of 70 eV with ion source temperature of 230°C, quadrupole temperature of 150°C, and transfer line temperature of 280°C.

Prior to analysis, the MS was autotuned to perfluorotributylamine (PFTBA) using already established criteria to check the abundance of *m/z* 69, 219, 502, and other instrument optimal and sensitivity conditions.

Analysis validation was conducted by running replicate samples in order to see the consistency of the constituent compound name, respective retention time, and molecular weight. These abundances were outputs from the *NIST 11 library search report* of the extracts constituents. Each compound identified via the NIST library search report has a corresponding mass spectrum showing the abundance of the possible numerous *m/z* peaks per compound.

### 2.9. Data Analysis

Data obtained were reported as mean ± standard error of mean (SEM) and appropriately analysed using one-way analysis of variance (ANOVA) with a subsequent Tukey's post hoc test (levels of significance were accepted at *p* < 0.05, *p* < 0.01, and *p* < 0.001). All statistical analysis was carried out using the software GraphPad PRISM (V.5.0).

## 3. Results and Discussion

**Figure 1:**
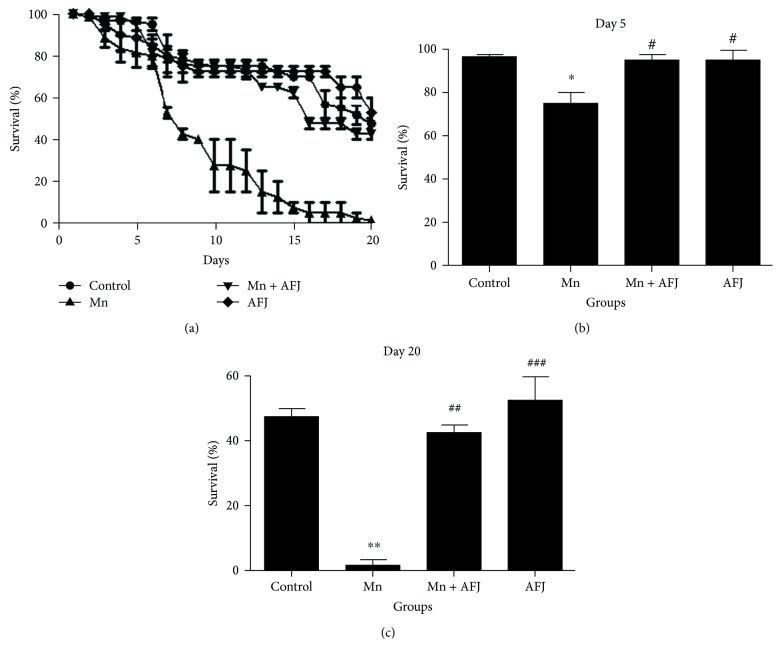
Effect of African Jointfir (AFJ) leaf alkaloid extract on: (a) survival, (b) day 5 survival rate, and (c) day 20 survival rate in Mn-induced toxicity in *Drosophila melanogaster*. Bars represent mean ± SEM. Mean values are significantly different at *P* < 0.05^∗^; *P* < 0.01^∗∗^ compared to control. Mean values are significantly different at *P* < 0.05^#^; *P* < 0.01^##^; *P* < 0.001^###^ compared to Mn-treated group.

**Figure 2:**
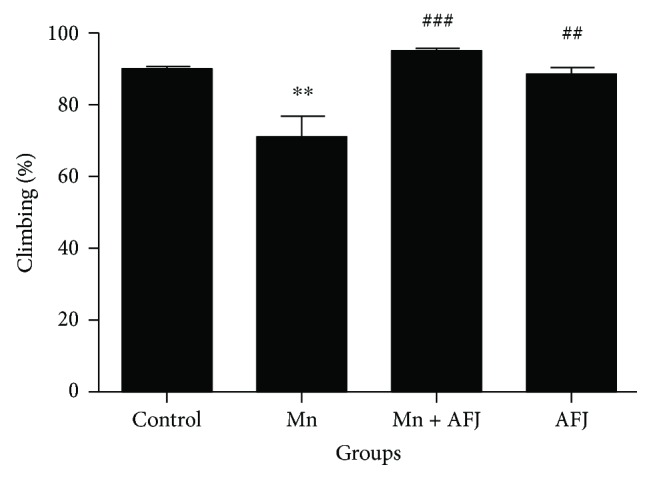
Effect of African Jointfir (AFJ) leaf alkaloid extract on: locomotor performance (climbing ability) in Mn-induced toxicity in *Drosophila melanogaster*. Bars represent mean ± SEM. Mean values are significantly different at *P* < 0.01^∗∗^ compared to control. Mean values are significantly different at *P* < 0.01^##^; *P* < 0.001^###^ compared to Mn-treated group.

In this present study, we assessed the mechanisms behind the protective ability of alkaloid extract of African Jointfir (AFJ) against manganese-induced neurotoxicity model in *D. melanogaster*. This study reveals that exposure of *D. melanogaster* to 10 mM of Mn for 20 days significantly (*P* < 0.05) reduced flies' survival rate (Figures 1(a)–1(c)) and locomotor performance (Figure 2). However, cotreatment with AFJ alkaloid extract significantly ameliorated the reduction in the survival rate and locomotor performance of Mn-induced flies. Previous studies have also reported that 10 mM Mn significantly reduced the survival rate and locomotor performance of *D. melanogaster*, and this could be associated with the cytotoxic effect of Mn [13]. Our findings also agree with earlier findings on the ability of plant alkaloid extracts to ameliorate Mn-induced impairment in the locomotor performance and survival rate of *D. melanogaster* [14, 38]. Furthermore, it has also been reported that plant alkaloid extract ameliorated Mn-induced neurotoxicity in rats [39, 40].

**Figure 3:**
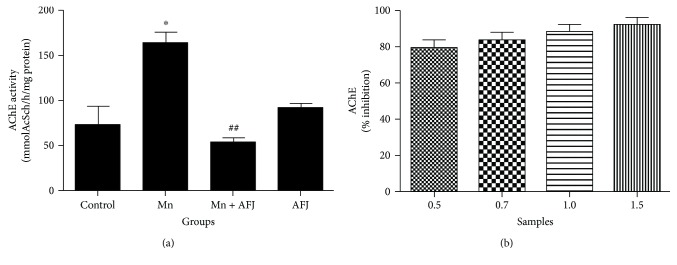
(a) Effect of African Jointfir (AFJ) leaf alkaloid extract on acetylcholinesterase (AChE) activity in Mn-induced toxicity in *Drosophila melanogaster.* (b) *In vitro* AChE inhibitory effect of AFJ leaf alkaloid extract. Bars represent mean ± SEM. Mean values are significantly different at *P* < 0.05^∗^ compared to control. Mean values are significantly different at *P* < 0.01^##^ compared to Mn-treated group.

Our findings showed that AFJ exhibit AChE inhibitory effect (*in vitro*) concentration dependently (Figure 3(b)) and also significantly ameliorate the elevation in the activity of AChE induced by Mn *in vivo* in Drosophila (Figure 3(a)). AChE catalyses the hydrolysis of acetylcholine to acetate and choline, thus regulating the cholinergic neuronal function [7]. Acetylcholine as a neurotransmitter is essential to regulate cognitive function, learning/memory, motor function, and locomotion [41]. Neurotoxic levels of Mn have been reported to impair the cholinergic system which is associated with Mn-induced impairments of motor function, locomotion, and cognitive dysfunction [41]. Therefore, as demonstrated in this present study, the increase in AChE activity after five days of exposure to Mn correlates to the significant decrease in their climbing abilities. The impairment observed in the climbing ability of the flies could be associated with a decrease in bioavailability of acetylcholine available for cholinergic neurotransmission [13, 42]. This phenomenon has been reported as a major risk factor in the development and progression of dementia [7]. Also, our findings are in agreement with earlier reports that short-term administration of Mn brought about a significant elevation in rat brain AChE activity [43, 44]. Another study revealed that exposure of rat to Mn caused an elevation in AChE activity in their serum and brain [45]. Consequently, the ability of AFJ alkaloid extract to ameliorate Mn-induced elevated AChE activity in flies, as well as increase the climbing ability of Mn-treated flies could suggest one of the mechanisms behind the protective effect of the extract against Mn-induced toxicity. In agreement with previous findings, anticholinesterase properties of plant extract have been substantially linked to their constituent phytochemicals including alkaloids; alkaloid extracts exhibit cholinesterase inhibitory properties *in vitro* [46, 47]. Konrath et al. reported that alkaloid extracts from Lycopodiaceae species from South America exhibited anticholinesterase activities *in vitro* and *in vivo* in the cerebral cortex, striatum, and hippocampus of rats following acute administration [48]. In addition, plant-derived alkaloids such as berberine, caffeine, evodiamine, isorhynchophylline, tetramethylpyrazine, and trigonelline have also been reported to show neuroprotective properties in various experimental models and are often associated with their anticholinesterase, antioxidant, and anti-inflammatory properties [49–54]. Therefore, AFJ alkaloid extracts also present a source of potent neuroprotective alkaloids. Generally, plant alkaloids are being promoted as drug leads for the management of neurodegenerative diseases owing to a number of their therapeutic mechanisms including anticholinesterase properties [55].

**Figure 4:**
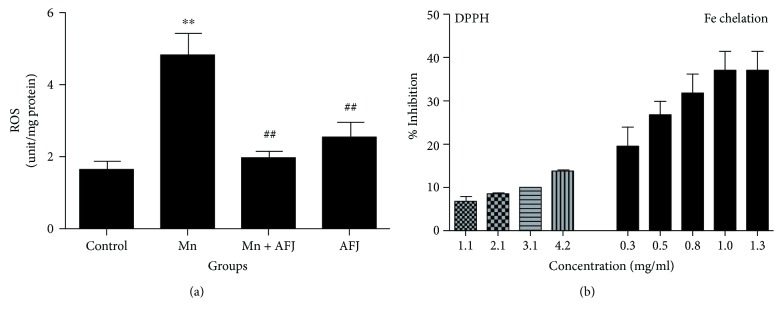
(a) Effect of African Jointfir (AFJ) leaf alkaloid extract on reactive oxygen species (ROS) level in Mn-induced toxicity in *Drosophila melanogaster.* (b) *In vitro* DPPH radical scavenging and Fe^2+^ chelating abilities of AFJ leaf alkaloid extract. Bars represent mean ± SEM. Mean values are significantly different at *P* < 0.01^∗∗^ compared to control. Mean values are significantly different at *P* < 0.01^##^ compared to Mn-treated group.

Earlier findings have implicated oxidative stress in Mn-induced neurotoxicity [13, 56, 57]. Oxidative stress, a consequence of ROS overload which cannot be effectively counteracted by the antioxidant defence mechanisms and hence, initiating cellular damage [58, 59]. In physiological systems, antioxidant enzymes including catalase, superoxide dismutase, and glutathione peroxidase are involved in preventing free radical overload and consequent oxidative stress. Results from this study showed that flies exposed to Mn exhibited a significant elevation in ROS level (Figure 4(a)) which is an indicator of oxidative stress. This is in agreement with earlier report on elevation of ROS level following Mn-induced toxicity in *Drosophila* [13, 60, 61]. However, cotreatment with AFJ alkaloid extract significantly ameliorated ROS level in Mn-intoxicate flies. This observation could be associated with the antioxidant properties of AFJ extract which can be further substantiated by their DPPH free radical scavenging and Fe^2+^ chelating abilities (Figure 4(b)). DPPH scavenging ability and Fe^2+^ chelation are two common assays carried out to assess the antioxidant potential of a test compound. Previous studies have shown that plant alkaloid extracts exhibit antioxidant properties which have been associated with their neuroprotective potentials [62]. The Fe^2+^ chelating ability of the extract in this study could be of significant therapeutic importance; first, excessive accumulation of Fe^2+^ could trigger chain redox reactions especially the Fenton reaction which can initiate the generation of cytotoxic ROS. Secondly, the chemical similarities between Mn and Fe have suggested that the neurotoxic effect of Mn is mediated by competing with Fe for “nonredox” domains in proteins [63]. Therefore, therapeutic agents with Fe chelating abilities are shown to be promising in ameliorating Mn-induced toxicity [41].

**Figure 5:**
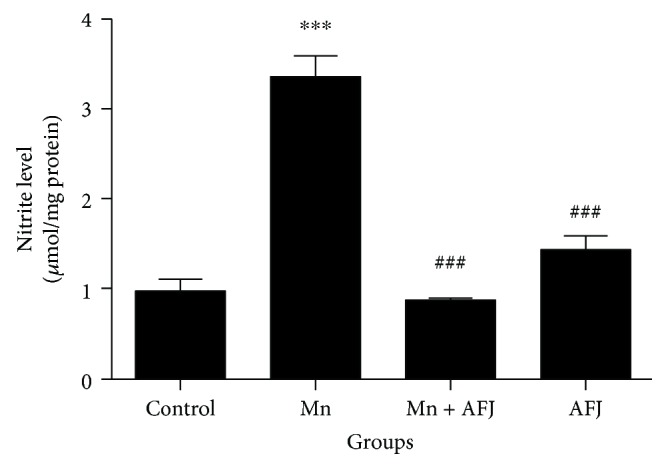
Effect of African Jointfir (AFJ) leaf alkaloid extract on: nitric oxide (NO) level in Mn-induced toxicity in *Drosophila melanogaster.* Bars represent mean ± SEM. Mean values are significantly different at *P* < 0.001^∗∗∗^ compared to control. Mean values are significantly different at *P* < 0.001^###^ compared to Mn-treated group.

**Figure 6:**
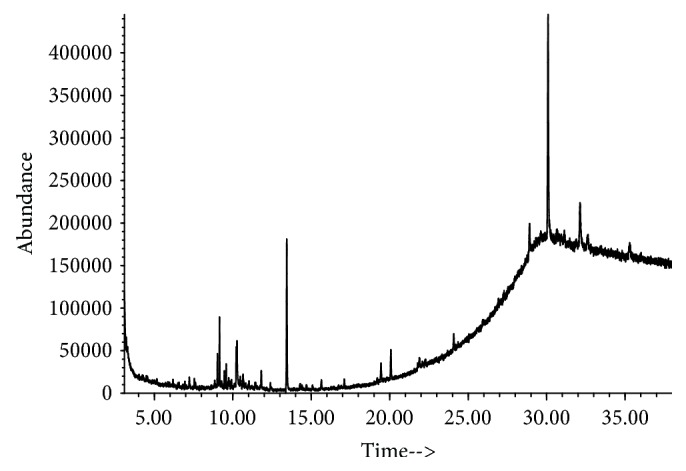
Total ion chromatogram (TIC) from GC-MS characterization of African Jointfir (*Gnetum africanum*) leaf alkaloid extract.

This study also shows that Mn was able to significantly elevate NO level in flies (Figure 5). This is similar to previous study which reported that Mn-induced toxicity caused elevation in NO level in rat brain cerebral cortex [2]. Nitric oxide is a diffusible signalling molecule in the nervous system of both vertebrates and insects [64]. In *D. melanogaster*, NO has been shown to mediate in cell proliferation and differentiation during fly developmental stages [65, 66]. NO also mediates in immune responses of the flies to pathogens and parasites [67, 68]. Generally, NO is reported to act as a proinflammatory mediator in which there is NO synthesis by inducible nitric oxide synthase (iNOS) that is significantly elevated [69]. Consequently, the ability of AFJ alkaloid extract to significantly ameliorate Mn-induced elevation in NO level in flies could suggest its potential anti-inflammatory property. In Figure 6 and Table 1, the result of the GC-MS characterization was presented, and the molecular structure of possible compounds in the extract were determined; this however demands further studies to fully elucidate the precise structural composition of constituent alkaloids in the extract for further pharmacological investigations.

Conclusion can therefore be drawn from this present study that alkaloid extract of AFJ was able to protect against manganese-induced toxicity by mechanisms including reducing oxidative stress, repressing AChE activity and proinflammatory mediator NO, and a consequent increase in climbing and locomotion activities in *Drosophila melanogaster.* Therefore, AFJ alkaloid extract could represent a novel source of neuroprotective compounds.

## Figures and Tables

**Table 1:** Possible compounds from GC-MS characterization of African Jointfir (*Gnetum africanum*) leaf alkaloid extract.

S/N	Possible structure	RT (min)^∗^	Molecular formula	MW^∗∗^	Exact mass (g/mol)	CAS number	EN^∗∗∗^
1	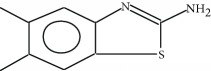	9.158	[C_9_H_10_N_2_S]	178	178.056469	29927-08-0	291167
2	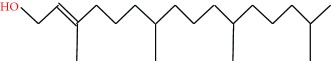	13.438	[C_20_H_40_O]	296	296.307917	150-86-7	375015

^∗^RT (min) = retention time (min); ^∗∗^MW = molecular weight (g/mol); ^∗∗∗^entry number in NIST 11 library.

## Data Availability

The data used to support the findings of this study are available from the corresponding author upon request.

## Abbreviations

AChE: Acetylcholinesterase
AFJ: African Jointfir
AD: Alzheimer's disease
DEPPD: *N,N*-diethyl-*para*-phenylenediamine
DPPH: 1,1-diphenyl–2 picrylhydrazyl
DMSO: Dimethyl sulfoxide
NO: Nitric oxide
PD: Parkinson's disease
ROS: Reactive oxygen species.
